# In Vivo Assimilation of CuS, Iron Oxide and Iron Oxide@CuS Nanoparticles in Mice: A 6-Month Follow-Up Study

**DOI:** 10.3390/pharmaceutics14010179

**Published:** 2022-01-13

**Authors:** Alberto Curcio, Aurore Van de Walle, Christine Péchoux, Ali Abou-Hassan, Claire Wilhelm

**Affiliations:** 1 Laboratoire Physico Chimie Curie, Institut Curie, CNRS, PSL Research University, 75005 Paris, France; alberto.curcio@univ-paris-diderot.fr (A.C.); aurore.vandewalle@univ-paris-diderot.fr (A.V.d.W.); 2 Laboratoire Matière et Systèmes Complexes (MSC), UMR 7057, CNRS, University of Paris, 75205 Paris, France; 3 INRAE, UMR 1313 GABI, MIMA2-Plateau de Microscopie Electronique, 78352 Jouy-en-Josas, France; christine.longin@inrae.fr; 4 PHysico-Chimie des Electrolytes et Nanosystèmes InterfaciauX, PHENIX, CNRS, Sorbonne Université, 75005 Paris, France; ali.abou_hassan@sorbonne-universite.fr

**Keywords:** CuS nanoparticles, iron oxide nanoparticles, magneto-plasmonic nanohybrids, metals bioassimilation, long-term biodistribution

## Abstract

Nanoparticles (NPs) are at the leading edge of nanomedicine, and determining their biosafety remains a mandatory precondition for biomedical applications. Herein, we explore the bioassimilation of copper sulfide NPs reported as powerful photo-responsive anticancer therapeutic agents. The nanoparticles investigated present a hollow shell morphology, that can be left empty (CuS NPs) or be filled with an iron oxide flower-like core (iron oxide@CuS NPs), and are compared with the iron oxide nanoparticles only (iron oxide NPs). CuS, iron oxide@CuS and iron oxide NPs were injected in 6-week-old mice, at doses coherent with an antitumoral treatment. Cu and Fe were quantified in the liver, spleen, kidneys, and lungs over 6 months, including the control animals, thus providing endogenous Cu and Fe levels in the first months after animal birth. After intravenous NPs administration, 77.0 ± 3.9% of the mass of Cu injected, and 78.6 ± 3.8% of the mass of Fe, were detected in the liver. In the spleen, we found 3.3 ± 0.6% of the injected Cu and 3.8 ± 0.6% for the Fe. No negative impact was observed on organ weight, nor on Cu or Fe homeostasis in the long term. The mass of the two metals returned to the control values within three months, a result that was confirmed by transmission electron microscopy and histology images. This bioassimilation with no negative impact comforts the possible translation of these nanomaterials into clinical practice.

## 1. Introduction

Metal sulfide nanoparticles (NPs) possess versatile optical and electronic properties, making them attractive for varied applications that include energy conversion and storage in solar cells [1,2,3], catalysts for industrial transformations, or carriers for drug delivery [4]. However, the toxicity of semiconductor NPs (e.g., ZnS and CdS) has been a limit to the range of possible applications, in particular in the biomedical area. For this reason, copper sulfide (CuS) NPs have received increased interest in recent years. In addition to their low toxicity [5], those nanostructures can be stimulated using near-infrared (NIR) light, an advantage in the biomedical field as it allows deep penetration into biological tissues, with minimal photodamage to the cells [4]. In particular, their absorption in both the 650–950 nm range (NIR I region) and in the second NIR region (1000–1700 nm, NIR II), where light absorption by tissues and photon scattering is lower, is valuable. This property is explored for photoacoustic imaging [6,7,8], as photothermal switch to activate signaling pathways [9], and for the photothermal ablation of cancer cells [10,11].

Another advantage of copper sulfide NPs is their ease of synthesis and their different stoichiometric compositions, with varying crystalline phases possible [12,13]. Among several designs, the synthesis of CuS hollow shell morphologies has been achieved. The synthesis of these hollow CuS can be implemented by a sacrificial templating method being at the same time green (water/70 °C), reproducible, and easily scaled up. The porous hollow structure obtained can hold a cargo for drug delivery [14,15] or surround any inorganic core (e.g., an iron oxide), which, in synergy with the physical plasmonic properties of CuS used in thermal therapies [16], can provide a dual-function to the nanostructures.

Otherwise, iron oxide NPs have been at the forefront of nanomedicine since its inception [17,18,19], mostly used for their magnetic properties that allow for magnetic resonance imaging [20,21] and thermal therapy via magnetic hyperthermia [22,23,24,25,26,27].

Recently, we developed hybrids combining a hollow CuS shell and a magnetic core, and confirmed their potential as multi-therapy agents [10]. In this case, the iron oxide@CuS hydrids were subjected to three stimuli: magnetic hyperthermia (MHT), photothermia (PTT), and photodynamic therapy (PDT), that altogether induced tumor regression via the production of heat and reactive oxygen species (ROS). We also explored the fate of such nanostructures in the biological environment in vitro, and reported low toxicity [28]. Interestingly, both CuS and iron oxide@CuS NPs could be biologically reshaped while preserving their photothermal efficiency.

A few studies reported the biological assimilation of copper sulfide NPs in vivo. Among them, Guo et al. showed 70 nm hollow CuS NPs are mostly taken up by the liver and kidney upon intravenous injection in mice, and eliminated within one month [29]. The biodistribution of CuS nanoplates was also explored in mice, and it was indicated they are mainly distributed in the liver, spleen, and lungs, without causing toxicity at the administrated dosage (5.5 mg/kg) [30]. CuS nanodots, much smaller in size (<6 nm), were mostly excreted intact within 24 h through the renal–urinary track [5]. Despite these studies, very little is known of the biological assimilation of copper sulfide NPs in vivo, even though it is an important requirement for all future medical applications [31]. By contrast, the in vivo bioassimilation of iron oxide NPs has been broadly studied [32,33,34,35,36,37], but never when combined with CuS. Importantly, Cu and, in a higher extent, Fe, are part of the metal ions naturally present in the mammalian metabolism. It has been shown that, within hours upon intravenous injection, iron oxide NPs are mainly captured by the liver, spleen, kidneys, and bone marrow [38,39,40,41]. The iron oxide cores can then be degraded, and the released iron joins the endogenous iron pool of the organism [42]. As an excess of iron can lead to cell damage and disease, understanding the handling of iron delivered to the organism upon administration of iron oxide NPs was central to their medical use. As similar copper-related toxicity can be reported in the case of copper homeostasis dysregulation, with copper accumulation potentially leading to Wilson disease, for instance [43], exploring the in vivo assimilation of Cu brought to the organism by CuS NPS appears to be equally important.

Herein, we provide a comparative study of the bioassimilation of copper and iron upon their injection as nanoparticles, either alone or combined as hybrids. The nanoparticles considered are flower-like iron oxides, CuS as a hollow shell, and the same CuS shell with iron oxide nanoflowers within the core (hybrid hollow iron oxide@CuS rattle-like structures). These nanoparticles were injected in vivo in mice, and a 6-month follow-up study was achieved to study the biological impact of their assimilation, in terms of organ weight, endogenous organ levels of iron or copper, iron and copper homeostasis and biodistribution in the long term.

## 2. Materials and Methods

### 2.1. Chemicals

Iron(II) chloride tetrahydrate (FeCl_2_·4H_2_O, 99%), sodium hydroxide (NaOH, 99.99%), diethylene glycol (DEG, 99%), N-methyldiethanolamine (NMDEA, 99%), nitric acid (HNO_3_, 70%), copper(II) nitrate hemi(pentahydrate) (Cu(NO_3_)_2_·2.5H_2_O, ≥99.99%), polyvinylpyrrolidone (PVP, Mw 55 kDa), poly(ethylene glycol) methyl ether thiol (PEG-SH, Mw 2 kDa) hydrazine hydrate (55%), ammonium sulfide solution ((NH4)_2_S, 20%), glutaraldehyde solution (25% in H_2_O), and sodium cacodylatetrihydrate (≥98%) were purchased from Sigma-Aldrich (Saint-Quentin-Fallavier, France). Iron(III) chloride hexahydrate (FeCl_3_·6H_2_O, 99%) and ethanol were obtained from VWR (Rosny-sous-Bois, France).

### 2.2. Nanoparticles Synthesis

The nanoparticles investigated here were previously reported by our team for their theranostics properties [10,28]. All details about the synthesis steps can be found there. In brief, polyol process was used for the preparation of magnetic nanoflowers (IONFs) [44]. At the end of the synthesis, the magnetic NPs were then collected magnetically, using a strong permanent magnet and washed several times before being oxidized into maghemite (γ-Fe_2_O_3_), flocculated in 10% nitric acid, washed twice with acetone, twice again with diethyl ether, and finally resuspended in water. For use as iron oxide NPs alone, and to ensure their aqueous stability, the nanoflowers were further functionalized by citrate anions absorption on their surface. For the CuS and IONFs@CuS hollow copper sulfide assemblies, the template sacrificial synthesis method was used, in presence or not of the iron oxide nanoflowers. At the end of the synthesis, sulfidation of the oxide shell was induced by the Kirkendall effect, before several washings by centrifugation and resuspension in Milli-Q water. The surface of all nanoassemblies was PEGylated by the addition of PEG-SH (final concentration 10 mg/mL) under shaking overnight at 4 °C and subsequent washing by centrifugation. In depth characterization of the nanoparticles obtained (HR-TEM, EELS, UV-Vis-NIR spectra, photothermal conversion parameter) are included in previous works [10,28]. Additional transmission electron microscopy characterization using a JEOL-1011 microscope operating at 100 kV is shown in Figure S1. NPs batches were prepared in 50 mL tubes, the reaction was reproducible and scalable, with shape morphology and optical properties of the NPs remaining similar between batches.

### 2.3. In Vivo IV Injection

Experiments involving animals were performed upon European-community-guideline requirements for animal care and use, registered to the ethics committee with the number APAFiS-CEB-15–2017, reference 2018020116521542, under the title “Etude de biodistribution et biodégradation de nanoparticules hybrides de cuivre et fer”. The 6-week-old Balb/C mice (weighting 25 g, on average) were injected with nanoparticles once. Doses of nanoparticles injected per mouse corresponded to 80 µg_Cu_ for CuS NPs, 110 µg_Cu_ and 20 µg_Fe_ for iron oxide@CuS NPs, and 70 µg_Fe_ for iron oxide NPs. At different days after injection (day 1, 3, 7, 30, 90, and 180), the mice were euthanized, and the organs were collected. Organs of control mice (without nanomaterial injection) were similarly collected at the same days.

### 2.4. Inductively Coupled Plasma (ICP) and Vibrating Sample Magnetometry (VSM) of Harvested Organs

Dosage of copper and iron was performed by inductively coupled plasma–mass spectroscopy (ICP–MS), at the platform quadrupole ICP–MS. Organs harvested at different days were digested in 200 µL of boiling 70% nitric acid for 1 h at 100 °C. The solutions obtained were diluted in filtered ultrapure water for ICP–MS analysis. Quantification of organ magnetization was measured using a PPMS device equipped with a vibrating sample magnetometer (VSM) option (Quantum Design, Les Ulis, France). The magnetic moment (in emu) of each sample was measured at 300 K, between 0 and 20,000 Oe.

### 2.5. Transmission Elecron Microscopy (TEM) of Harvested Organs

Harvested organs were rinsed and divided into pieces (1 mm^3^ each). Dissected samples were then fixed with 4% glutaraldehyde in 0.1 M cacodylate buffer for 2 h, treated with a microwave tissue processor, contrasted with 0.5% oolong tea extract (OTE), post-fixed in 1% osmium tetroxide, and embedded in epoxy resins. Samples were sectioned into ultrathin slices (70 nm) and counterstained with lead citrate. TEM images were taken with a Hitachi HT 7700 (Hitachi, Tokyo, Japan) operated at 80 kV.

## 3. Results and Discussion

### 3.1. Quantification of Endogenous Copper and Iron Mass in Liver, Spleen, Kidneys and Lungs over 6 Months

**Figure 1:**
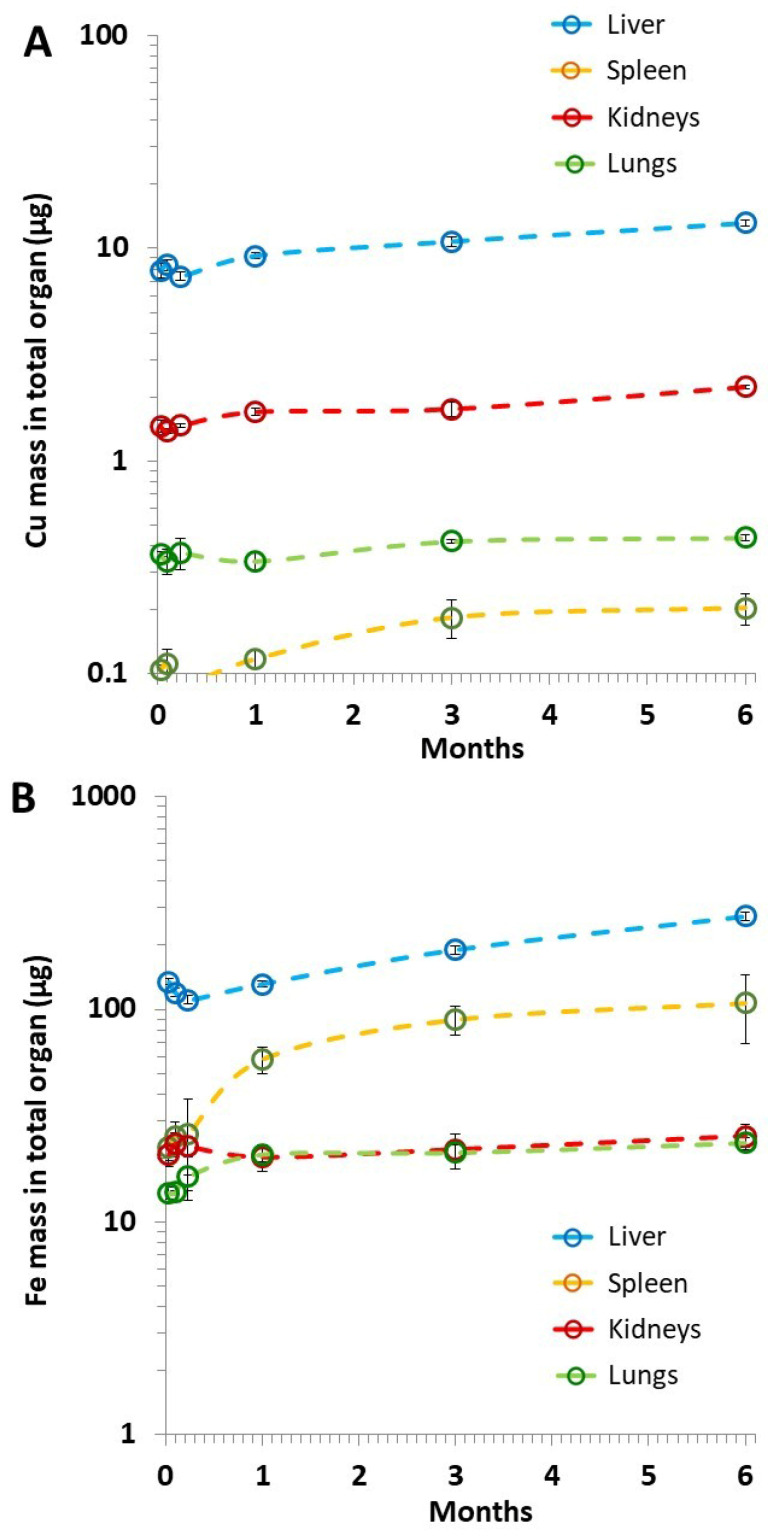
Quantification of endogenous Cu (**A**) and Fe (**B**) mass per organ, in the control mice. The mice were 6-weeks old at the beginning (month 0) and the level of endogenous Cu and Fe was followed via inductively coupled plasma (ICP) analyses over 6 months (*n* ≥ 4 for each condition). Error bars correspond to the SEM (standard error of the mean).

Quantification of endogenous iron and copper mass was performed on the control mice. The mice were 6-weeks old at the beginning of the study and the level of these two metals was followed over 6 months in the liver, spleen, kidneys, and lungs. Results are displayed in Figure 1. These initial data show a progressive increase in mass per organ of both copper (Figure 1A) and iron (Figure 1B) over time, as well as a clear difference in their amount at a given time, with the mass of iron always being higher. For instance, at 6 months, the mass of endogenous iron was 20-times higher in the liver, 15-times higher in the kidneys, 50-times higher in the lungs, and 500-times higher in the spleen, compared with the endogenous copper. Such iron accumulation in the spleen certainly echoes the spleen’s role in maintaining a healthy red blood cell population and storing iron [45]. Herein, we also observed a strong increase in the spleen iron content with age (Figure 1B). Iron accumulation was previously reported in 25-week old mice [46] but the quantification with age, as achieved here between 6-week and 6-month old mice, was missing, to the best of our knowledge. Endogenous iron and copper can also be expressed in terms of mass per gram of organ, with values at 6 months in the range of 8 µg of Cu and 175 µg of Fe per gram of liver, 1.3 µg of Cu and 700 µg of Fe per gram of spleen, 4 µg of Cu and 45 µg of Fe per gram of kidneys, and 2.5 µg of Cu and 145 µg of Fe per gram of lungs.

### 3.2. Quantification of Copper and Iron Mass in Organs after Intravenous Injection of the CuS and Iron Oxide Nanoparticles

**Figure 2:**
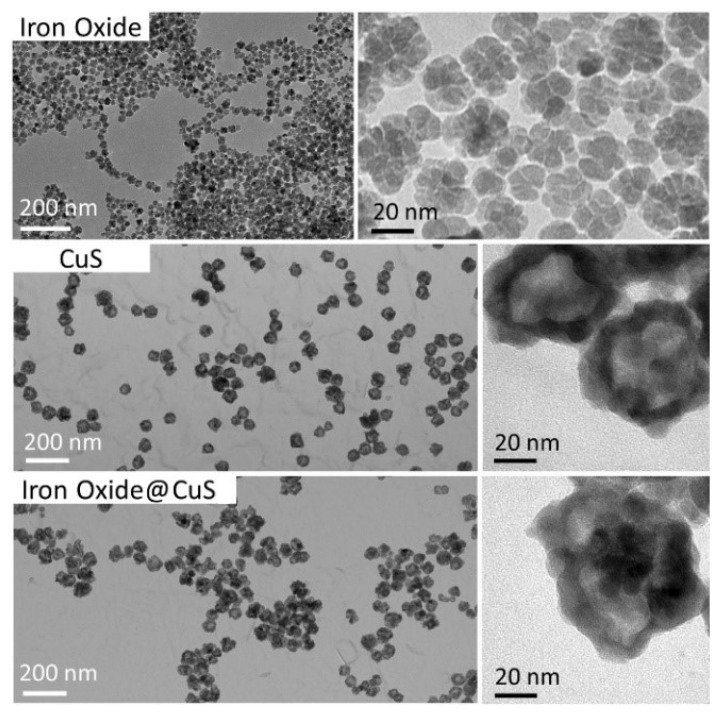
Transmission electron images of CuS NPs and/or iron oxide NPs. Iron oxide flower-like NPs (**Top**). Hollow copper sulfide (CuS) nanoassemblies (**Middle**). Rattle-like nanohybrids composed of an iron oxide nanoflower core surrounded by a hollow CuS shell (**Bottom**).

Transmission microscopy (TEM) images of the nanoparticles before injection are shown in Figure 2 and display the iron oxide flower-like NPs, the hollow copper sulfide (CuS) nanoassemblies, and the nanohybrids made of a rattle-like iron oxide nanoflowers incorporated inside hollow CuS. Diameter measurements based on the TEM imaging indicated an average diameter of 57.4 ± 7.8 nm for CuS NPs, 23.6 ± 3.5 nm for the iron oxide NPs alone or within the CuS core, and 65.0 ± 8.6 nm for the entire nanohybrid (iron oxide@CuS). In addition, both CuS and iron oxide@CuS NPs had same hydrodynamic diameter at about 90 nm, stable in cellular media.

The nanoparticles were injected into 6-week-old mice at intravenous doses of copper and iron, set at 80 µg_Cu_/mouse for CuS, 110 µg_Cu_/mouse and 20 µg_Fe_/mouse for iron oxide@CuS, and 70 µg_Fe_/mouse for iron oxide. The choice of these doses was motivated by the anticancer photothermal therapy application that CuS-based nanomaterials have recently been developed for. For obtaining an efficient photothermal action, intratumoral injections were performed within the range of 10 to 50 µg of Cu [10], making it necessary to assess the impact of such a ranged dose of copper on distant organs, in order to understand the systemic effects of treatment on the whole body in case of nanomaterial release.

**Figure 3:**
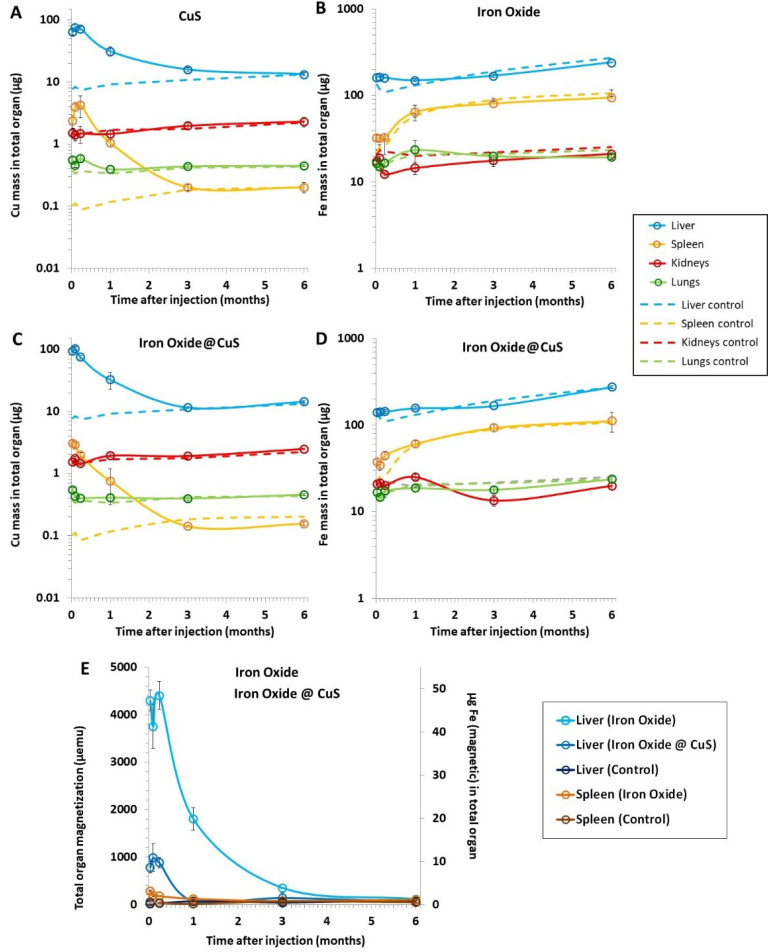
Follow-up of copper and iron mass into the organs after NP administration. (**A**–**D**) The evolution of Cu and Fe levels into the liver, spleen, kidneys, and lungs of mice (6-weeks old at the time of injection) was assessed via ICP after injection of (**A**) CuS NPs, (**B**) iron oxide NPs, or (**C,D**) iron oxide@CuS NPs. (**E**) Levels of magnetic iron were also measured for the iron oxide-based NPs, using vibrating sample magnetometry (VSM). The magnetic moment at saturation, in emu, is shown on the primary axis, and the corresponding mass of magnetic iron has been calculated and is shown on the secondary axis. Dotted lines represent the linear regressions of the control values. Error bars correspond to the SEM (*n* ≥ 5 for each condition).

Upon the NP’s intravenous injection, the mass of copper and iron was quantified over time in the liver, spleen, kidneys, and lungs, and compared with the endogenous levels obtained in the control mice. The results are presented in Figure 3. It was immediately evidenced that there was no difference in Cu and Fe levels in the long term between the injected mice and the controls, as demonstrated by the plain and dotted lines all superposed at 3–6 months (Figure 3A–D). Thus, no overdose of metal lasted within the time. It was also obvious that Cu and Fe levels in the kidneys and lungs remained almost identical to the control values for the entire experimental period, meaning that the nanoparticles were barely integrated into these organs. On the contrary, for the copper-based nanomaterials (i.e., CuS and iron oxide@CuS), the Cu levels largely increased in the liver and spleen, in comparison to the controls (Figure 3A,C). This was particularly spectacular in the spleen, with a more than 50-times increase compared with the endogenous levels. A 10-times increase was measured for the liver. For the iron-based nanomaterials (i.e., iron oxide and iron oxide@CuS, Figure 3B,D), slight variations were perceived, but they remained very low in regard to the high endogenous levels. Indeed, the endogenous level of iron in the liver was above 100 µg, and the amount of injected iron was 70 µg for iron oxide NPs and 20 µg for iron oxide@CuS NPs. Nevertheless, it is possible to circumvent these limitations by using magnetometry (Figure 3E). Only the superparamagnetic iron was then measured, stemming only from the magnetic NPs injected, inexistent for the control organs, thus facilitating the distinction between endogenous iron and exogenous iron coming from the nanoparticles. The results showed a clear increase in magnetic iron into the liver upon injection of iron oxide NPs and iron oxide@CuS NPs, these values being maximal at day 7, and returning to the control levels in the long term (after 3–6 months). A slight increase was also observed in the spleen in the first days upon injection.

### 3.3. Biodistribution of Copper and Iron into the Organs over Time

**Figure 4:**
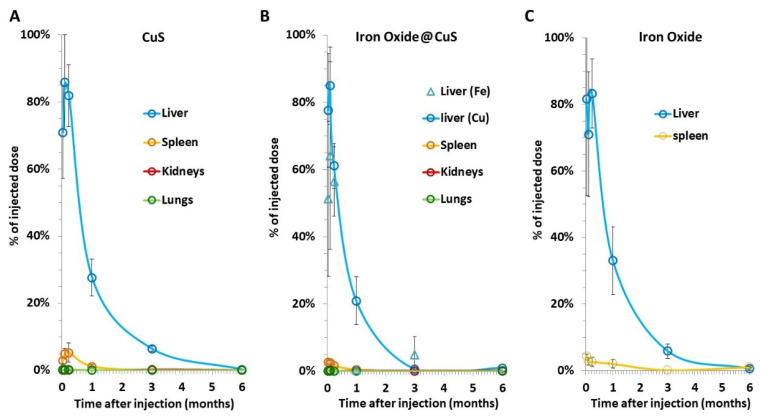
Percentage of nanomaterial-injected dose detected in the organs over time. This dose was calculated for (**A**) copper only in CuS NPs; (**B**) both copper and iron in iron oxide@CuS NPs; and (**C**) iron only in iron oxide NPs. Results were extrapolated from ICP data for copper and VSM data for iron. Error bars correspond to the SEM (*n* ≥ 5 for each condition).

To retrieve the NPs biodistribution in the assessed organs (liver, spleen, kidneys, and lungs) and over time, results were calculated as the percentage of the injected dose (Figure 4). These data were extrapolated from the ICP measurements for copper (CuS and iron oxide@CuS) and from the magnetism values for iron (iron oxide@CuS and iron oxide). For both copper and iron, about 80% of the injected dose was located in the liver in the first days (1–7), and 3–5% was found in the spleen, both robustly regardless of the nanoparticles.

### 3.4. No Long-Term Impact on Organ Weights, Endogenous Cu and Fe Levels, and Expression of Inflammation- and Fe/Cu Metabolism-Related Genes

The impact of CuS, iron oxide, and iron oxide@CuS NPs on the mice’s metabolism was then assessed. First, the weight of all studied organs was measured over time and compared between the control and the injected mice as a marker of overall toxicity (Figure S2). The results showed that neither of the nanoparticles had an impact on organ weight in the 6 months following injection. The mice injected with single metal NPs (CuS only or iron oxide only) were then used as a means of evaluating the effects on the endogenous homeostasis of the opposite metals (Figure S3). Copper mass was thus followed after the injection of iron oxide, and no difference between the control and injected mice was identified (Figure S3A). Similarly, iron mass remained analogous to the control levels after CuS injection (Figure S3B). This indicates that the NP’s administration had no effect on the endogenous copper or iron levels. Finally, the expression of gene coding for the proteins involved in iron and/or copper homeostasis (ferritin, ferroportin, and ceruloplasmin) as well as inflammation (iNOS, TNFα, and IL6) were quantified in the liver, where the highest dose of nanomaterial was directed (Figure S4). The resulted showed no significant effect of the injections on inflammatory genes, either in the short term (days 1–3–7) or the long term (months 1–3). For the metal-related genes, only a slight decrease in the expression of the ferroportin and ceruloplasmin was observed in the initial days (1–3–7) for iron oxide@CuS NPs and iron oxide NPs and, in the long term, no significant effect was detected. These data indicate that the administered NPs had no impact on the overall metabolism of the mice in the long term. This correlates nicely with previous analyses performed in vitro that showed an adaptation of the cellular metabolism to these nanomaterials with no toxicity reported, even at higher doses [28].

### 3.5. Structural Monitoring of the Administerd NPs by Transmission Electron Microscopy (TEM)

**Figure 5:**
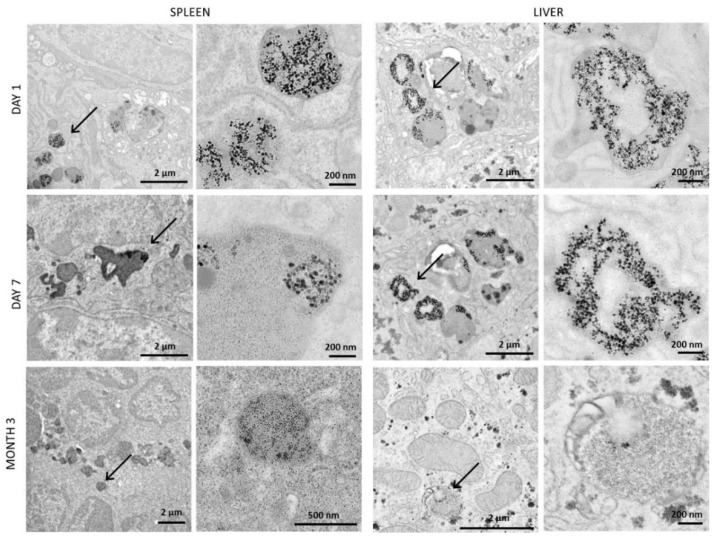
TEM images of the spleen and the liver following injection of iron oxide flower-like NPs into mice. Images taken at days 1 and 7 show intact iron oxide nanoflowers in both organs, as indicated by the arrows. By contrast, 3 months after injection, only very few intact iron oxide NPs remain, while numerous 6 nm dark-contrast dots, typical of ferritin proteins, can be observed (arrows).

**Figure 6:**
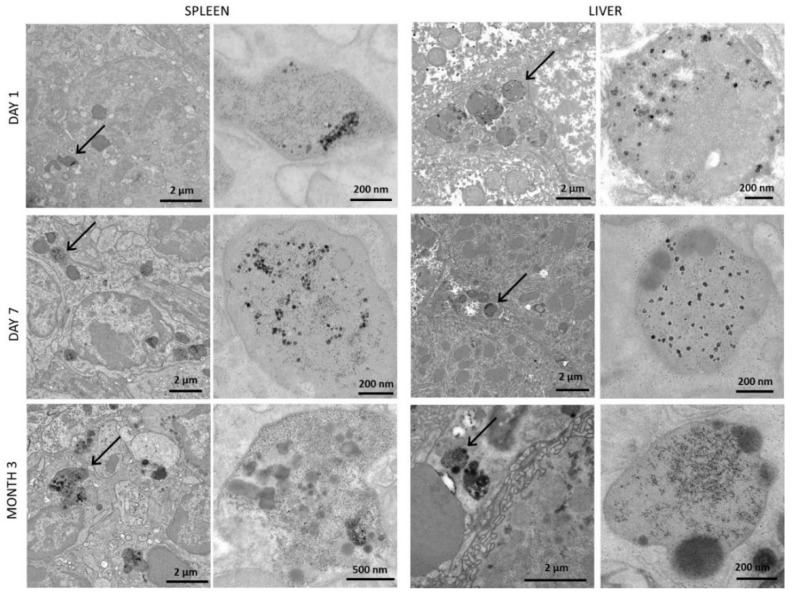
TEM images of the spleen and the liver of mice after administration of iron oxide@CuS NPs. The hybrids were easily detected in the images via their iron oxide core, more contrasted than the CuS assembly. This CuS shell can sometimes be noticed, such as in the zoomed-in image of the liver at day 1 where they appear as halos around the iron oxide core. At days 1 and 7, intact iron oxide@CuS can be observed in both organs, while none remain after 3 months.

**Figure 7:**
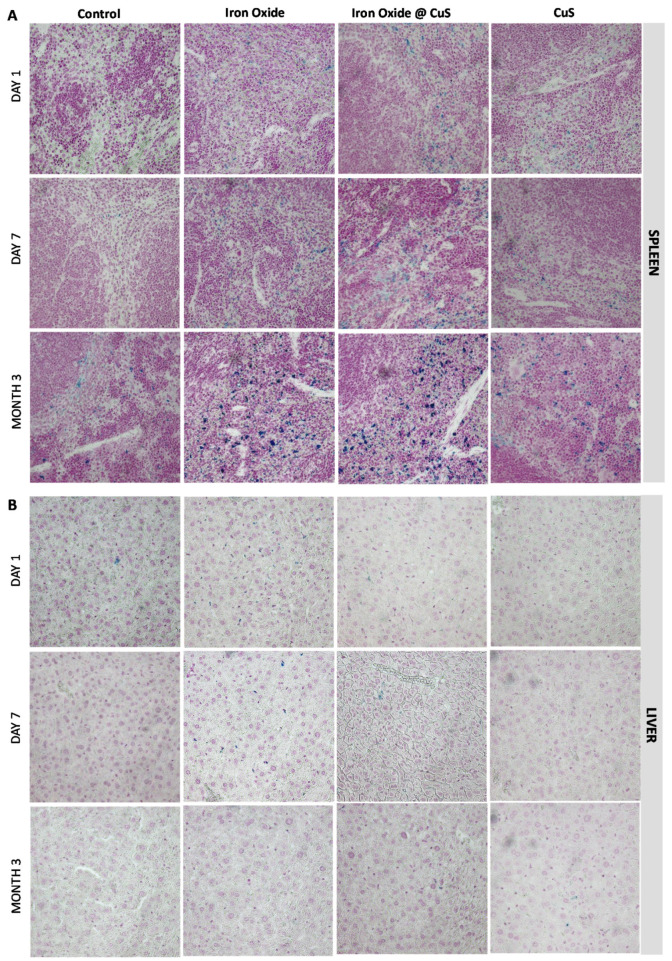
Prussian blue staining: Histological images of the spleen (**A**), and liver (**B**), stained for iron at day 1, day 7, and month 3. The Perls Prussian blue staining was used to detects iron in blue, while the cells appear pink.

As the quantification of Cu and Fe levels and the biodistribution analyses indicated that the nanomaterials were mainly directed into the liver and the spleen after injection, TEM imaging of those organs was achieved 1 and 7 days after injection, when a significant increase in Cu and Fe mass per organ was observed, and 3 months after injection when the mass returned to the control values. Images were first taken in control mice and showed numerous dark-contrast dots, about 6 nm in size (Figure S5). These structures are typical of the ferritin protein, primarily responsible for iron storage, which appeared in very large numbers in the spleen (Figure S5A) and in slightly decreased numbers (but remaining high) in the liver (Figure S5B). These results can be correlated with the important mass of endogenous iron naturally present in these organs. In parallel to the control mice, TEM images were taken upon injection of iron oxide NPs only, at day 1, day 7, and month 3 (Figure 5). In this case, the flower shape of the injected NPs and the dark contrast coming from their iron oxide composition allowed them to be easily recognized. The results showed numerous intact iron oxide flower-like NPs in both the spleen and the liver at days 1 and 7, while they were mostly absent 3 months after injection (see Figure S6 for additional images). These results are in agreement with previous data indicating an increased iron mass in the first days following injection and a return toward basal levels after 3 months. At 3 months, numerous dark dots, typical of ferritin proteins, were observed in the surrounding areas of the few remaining iron oxide NPs. This correlates with the progressive increase of iron mass in these organs over time, this metal being stored in the ferritin proteins. Regarding the origin of the stored iron, it might either be endogenous or coming from the degradation of the nanoflowers. In any case, the iron metabolism in these organs has been adjusted and its homeostasis restored, as indicated previously by the mass of iron that returns to control values 3 months after injection (Figure 2B). Presence of the hybrids (iron oxide@CuS) was also monitored over time, using TEM imaging (Figure 6). In this case, the nanomaterials could be identified via the high contrast of their iron oxide core. In some cases, the CuS shell was also noticeable; it appeared as a halo around the flower-like iron oxide core (see Figure 6, day 1 in the liver, and additional images in Figure S7). Intact hybrids were observed in both the spleen and the liver at days 1 and 7, as well as an increased amount of ferritin-like dots at day 7. At 3 months, no intact hybrid could be detected, although numerous ferritin-like structures were present. Again, images correlated with the increase in Cu and Fe mass observed in the first days upon injection, followed by the return to endogenous levels after 3 months. In the case of CuS, the low contrast of the CuS nanomaterials did not allow their accurate monitoring via TEM, as it was almost impossible to distinguish the nano-objects. The images taken are shown in Figure S8. Histological analyses of the same two organs, stained for iron with Prussian blue, were further achieved (Figure 7). Logically, they showed an increased amount of iron in the spleen when compared with the liver, which correlates with the µg_Fe_/g_organ_ values detailed previously. Iron detection also increased with mice aging, which reflects the homeostasis of endogenous iron. Finally, an increase in iron was detected after injection of iron oxide NPs and iron oxide@CuS NPs, in agreement with their biodistribution.

## 4. Conclusions

The in vivo long-term fate of Cu- and Fe-based NPs was assessed in terms of Cu and Fe organ contents over 6 months, together with structural electron microscopy of the materials in the liver and spleen. The Cu and Fe values over animal-aging (from 6-weeks to 6-months) were indicative of a rich homeostasis of Cu and Fe in mammals, in particular with a massive increase in the spleen. The NPs doses injected were chosen to mimic the exposure on healthy distant organs after an antitumoral treatment. Importantly, the low impact of the three NPs during their assimilation was demonstrated. The weight of the organs overtime (liver, spleen, kidneys, and lungs) was not modified by the injection, neither were the endogenous levels of iron or copper in these organs (following CuS or iron oxide injection, respectively). No impact was reported on inflammatory gene expression in the liver, where 80% of all nanoparticles are directed within the initial days. Only a slight dysregulation of genes involved in iron and copper homeostasis was observed in the first days upon injection, as well as an increased iron and copper mass, which both returned to control levels within 3 months. Concerning the NP’s biodistribution, because the Cu endogeneous levels remained highly inferior to the Fe ones, it was possible to perform an accurate Cu-derived dosage on all organs. For Fe-derived measurements, only the magnetometric ones could infer the biodistribution. Remarkably, all converged towards 80% of the injected materials in the liver for the first 7 days, and a renormalized to endogeneous levels at months 1–3. Finally, TEM observations showed intact NPs within the liver and spleen at days 1 and 7, and close to none were present at 3 months. Large numbers of ferritin proteins, responsible for iron storage, were revealed. The similar biological handling and absence of a long-term impact of CuS, iron oxide, and iron oxide@CuS NPs confirm their potential as biomedical agents, in particular as thermal agents combining magnetic hyperthermia and photothermal properties [10].

## Data Availability

The data that support the findings of this study are available from the corresponding author and in the Supplementary Materials file.

## Supplementary Materials

The following supporting information can be downloaded at: https://www.mdpi.com/article/10.3390/pharmaceutics14010179/s1, Figure S1: TEM imaging of iron oxide flowers-like NPs, CuS NPs and iron oxide@CuS NPs; Figure S2: Organ weight for control and injected mice; Figure S3: Effect of the nanomaterials on endogenous copper and iron levels; Figure S4: Expression of metal- (A–C) and inflammation-related (D–F) genes, in the liver; Figure S5: Transmission Electron Microscopy (TEM) images of control mice; Figure S6: TEM images of the liver (A) and the spleen (B) of mice injected with 70 µg Fe from iron oxide nanoflowers; Figure S7: TEM images of the spleen (A) and the liver (B) of mice injected with 110 µg Cu/20 µg Fe from iron oxide@CuS NPs; Figure S8: TEM images of the spleen (A) and the liver (B) of mice injected with 80 µg Cu from CuS NPs.
