# Supplementary material for: In Vivo Assimilation of CuS, Iron Oxide and Iron Oxide@CuS Nanoparticles in Mice: A 6-Month Follow-Up Study
Source: Pharmaceutics. 2022 Jan 13;14(1):179. doi: 10.3390/pharmaceutics14010179 (PMC8780448; doi:10.3390/pharmaceutics14010179)
Supplement: Supplementary file 1 [file pharmaceutics-14-00179-s001.zip › pharmaceutics-1532577-supplementary.pdf]

Supplementary Material: In Vivo Assimilation of CuS, Iron Oxide and Iron Oxide@CuS Nanoparticles in Mice: A 6-Month Follow-Up Study

Alberto Curcio, Aurore Van de Walle, Christine Péchoux, Ali Abou-Hassan and Claire Wilhelm

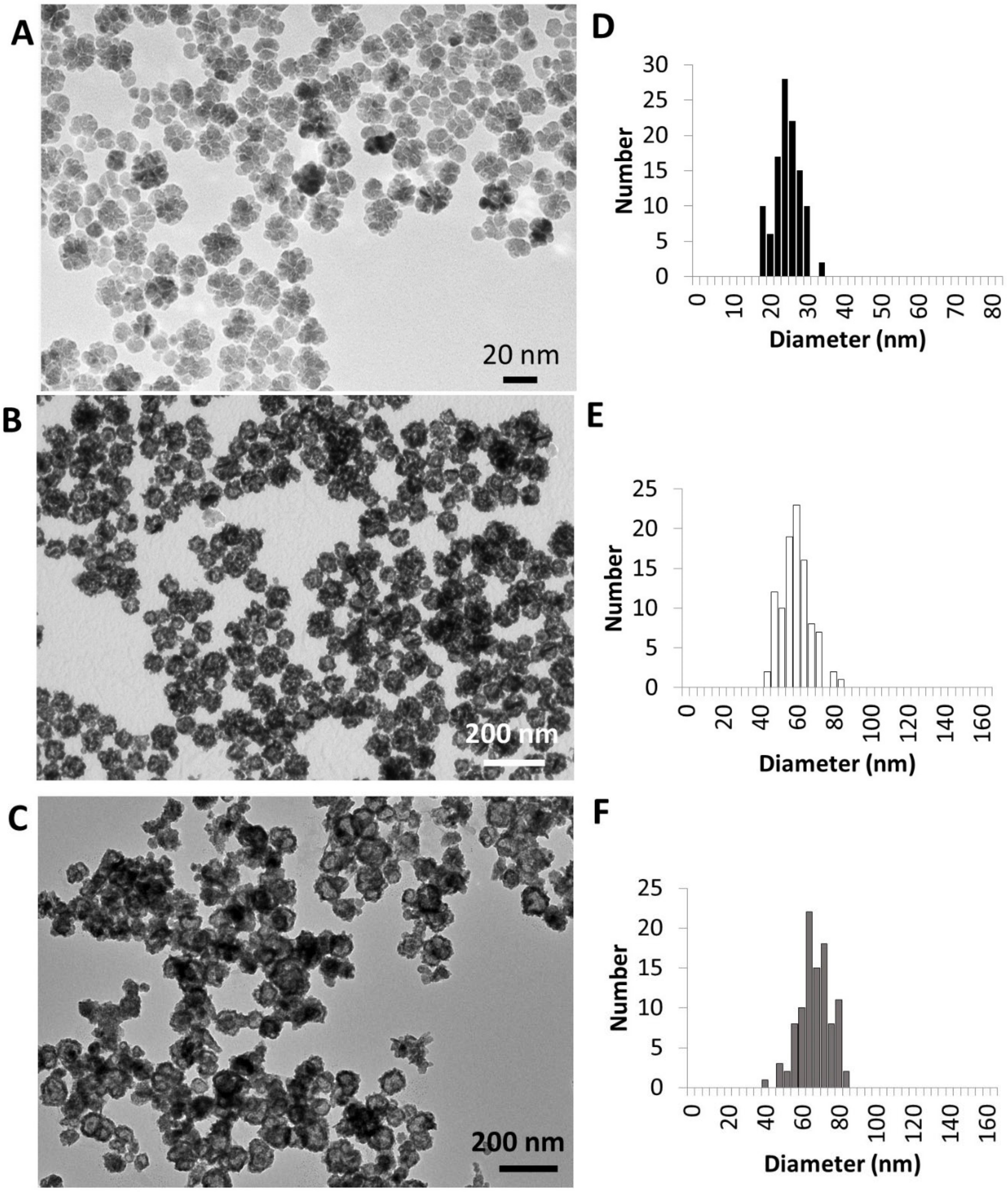

Figure S1. Transmission Electron Microscopy (TEM) imaging of iron oxide flowers-like NPs (**A**), CuS NPs (**B**) and Iron Oxide@CuS NPs (**C**). Diameters were measured over 100 nanoparticles, with distribution shown in (**D**) for iron oxide nanoflowers, (**E**) for CuS, and (**F**) for Iron Oxide@CuS.

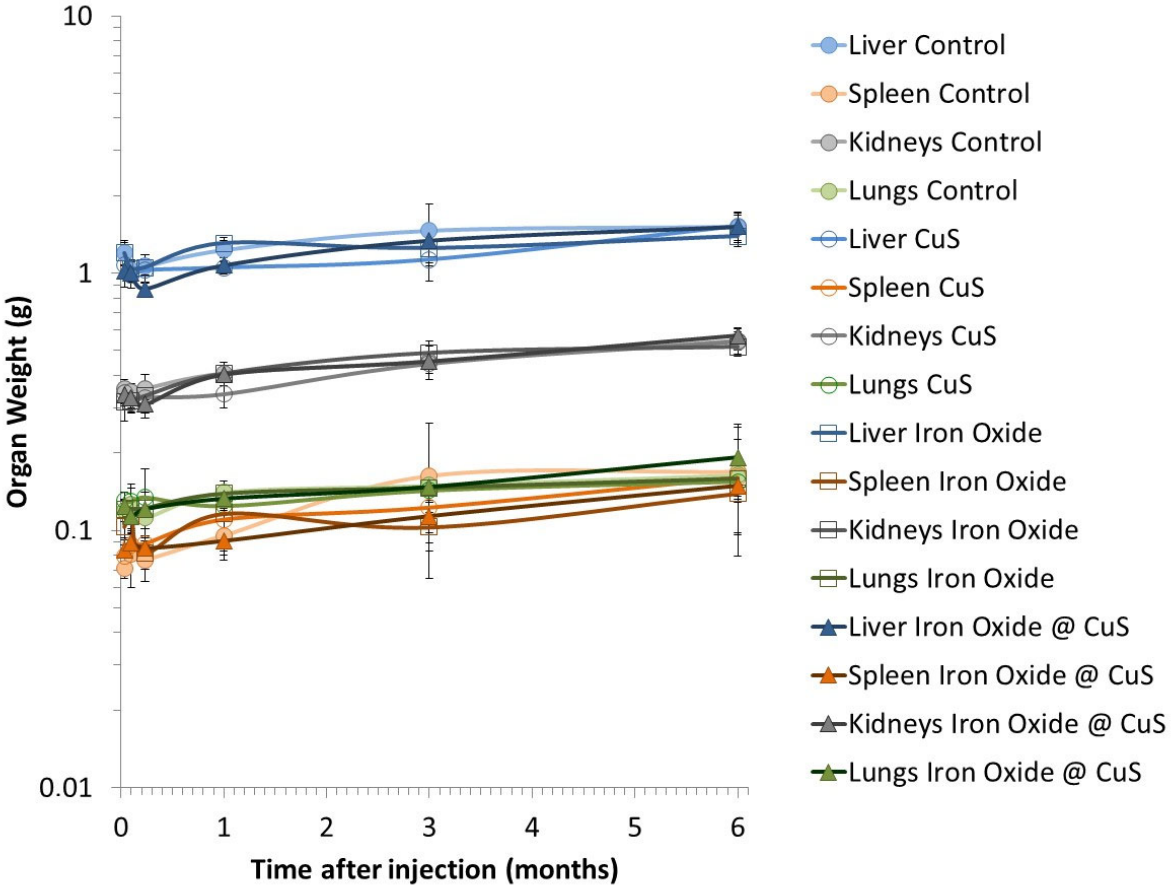

Figure S2. **Organ weight for control and injected mice**. Liver (blue), spleen (orange), kidneys (grey), and lungs (green) were weighted (in grams) at the different time points after the nanoparticles injection (control, CuS, Iron Oxide, Iron Oxide@CuS). Each data point represents an average extrapolated from at least 6 mice.

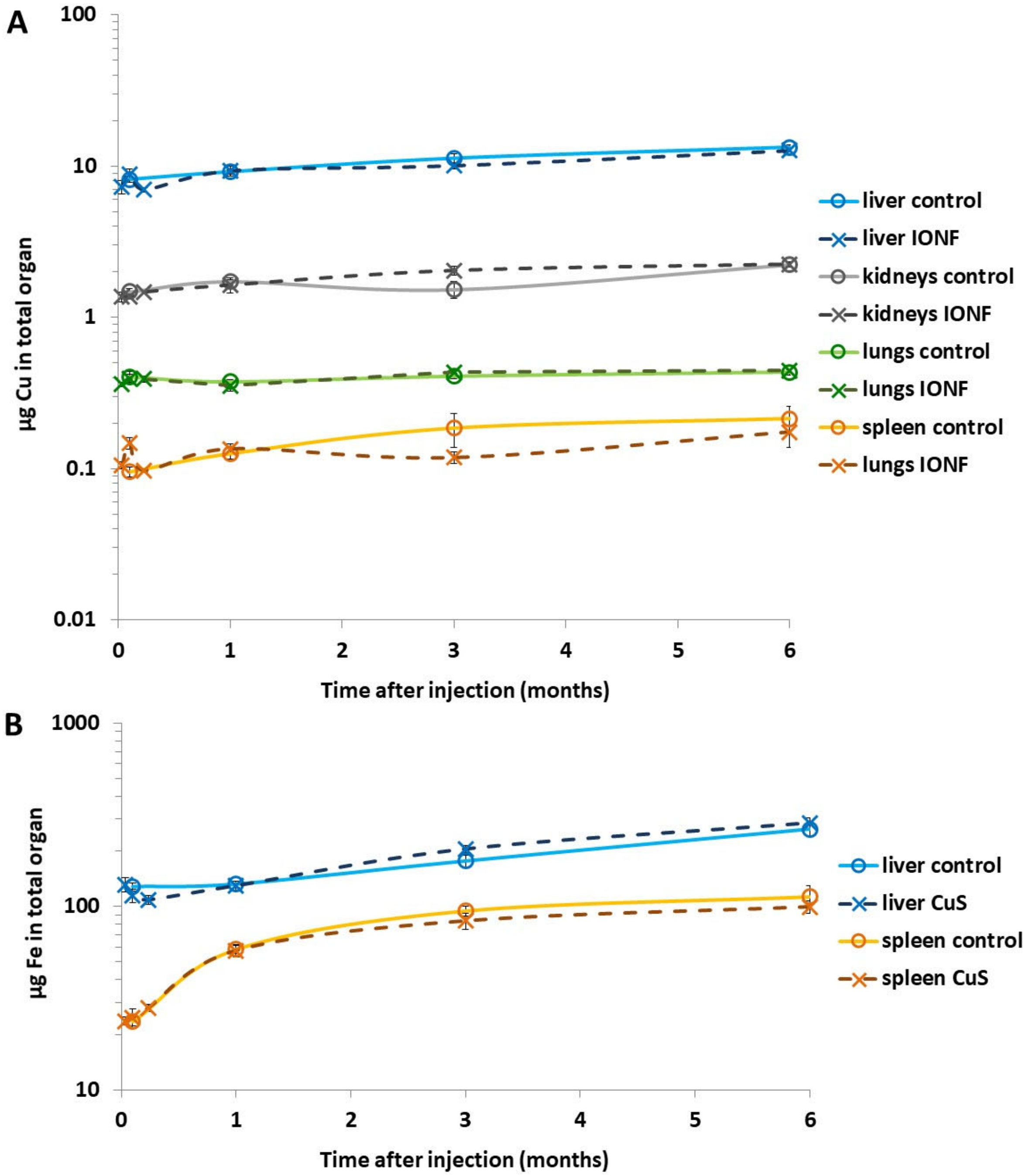

Figure S3. **Effect of the nanomaterials on endogenous copper and iron levels.** The mass of copper and iron is compared between control mice and mice injected with Iron Oxide nanoparticles only (**A**) or CuS nanoparticles only (**B**), respectively.

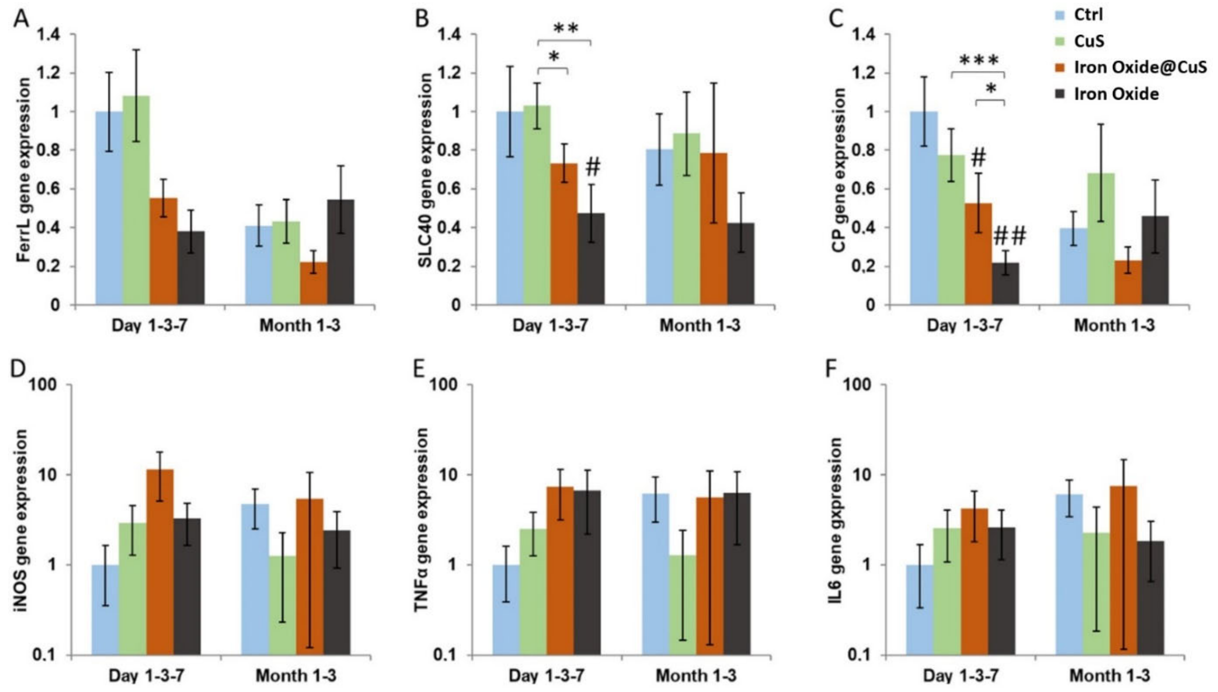

Figure S4. Expression of metal- (A–C) and inflammation-related (D–F) genes, in the liver. Gene expression levels have been quantified for (**A**) ferritin L (FerrL), (**B**) ferroportin (SLC40), (**C**) ceruloplasmin (CP), (**D**) nitric oxide synthase (iNOS), (**E**) tumor necrosis factor-alpha (TNFα), and (**F)** interleukin 6 (IL6). The expression is averaged for days 1-3-7 and months 1-3, and expressed relatively to the averaged control for days 1-3-7. Values are presented as mean and standard error of the mean. Significant differences between groups was determined using a Tukey's test in one-way analysis of variance (ANOVA). Stats and graphs were generated in Excel. #significance relative to the control and * significance between the nanomaterials, with * p <0.05, ** p <0.005, *** p <0.001 and # p <0.05, ## p <0.005.

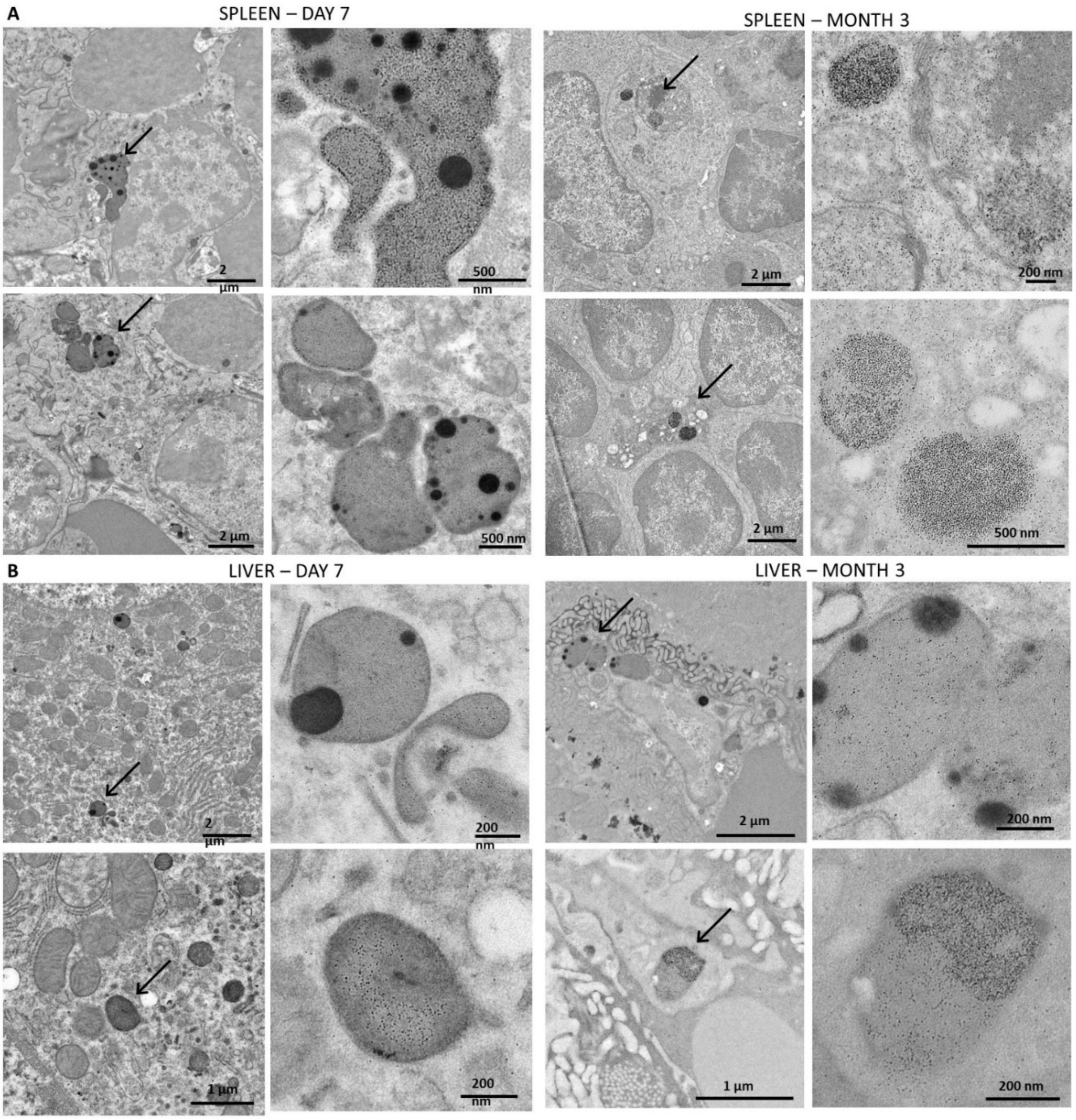

Figure S5.: Transmission Electron Microscopy (TEM) images of control mice, at day 7 (7-weeks old, left) and at month 3, in spleen (**A**) and in liver (**B**). Large fields are shown on the left, with areas indicated by a black arrow then zoomed in to the right. These areas contain numerous 6-nm dark contrast dots, typical of the ferritin protein.

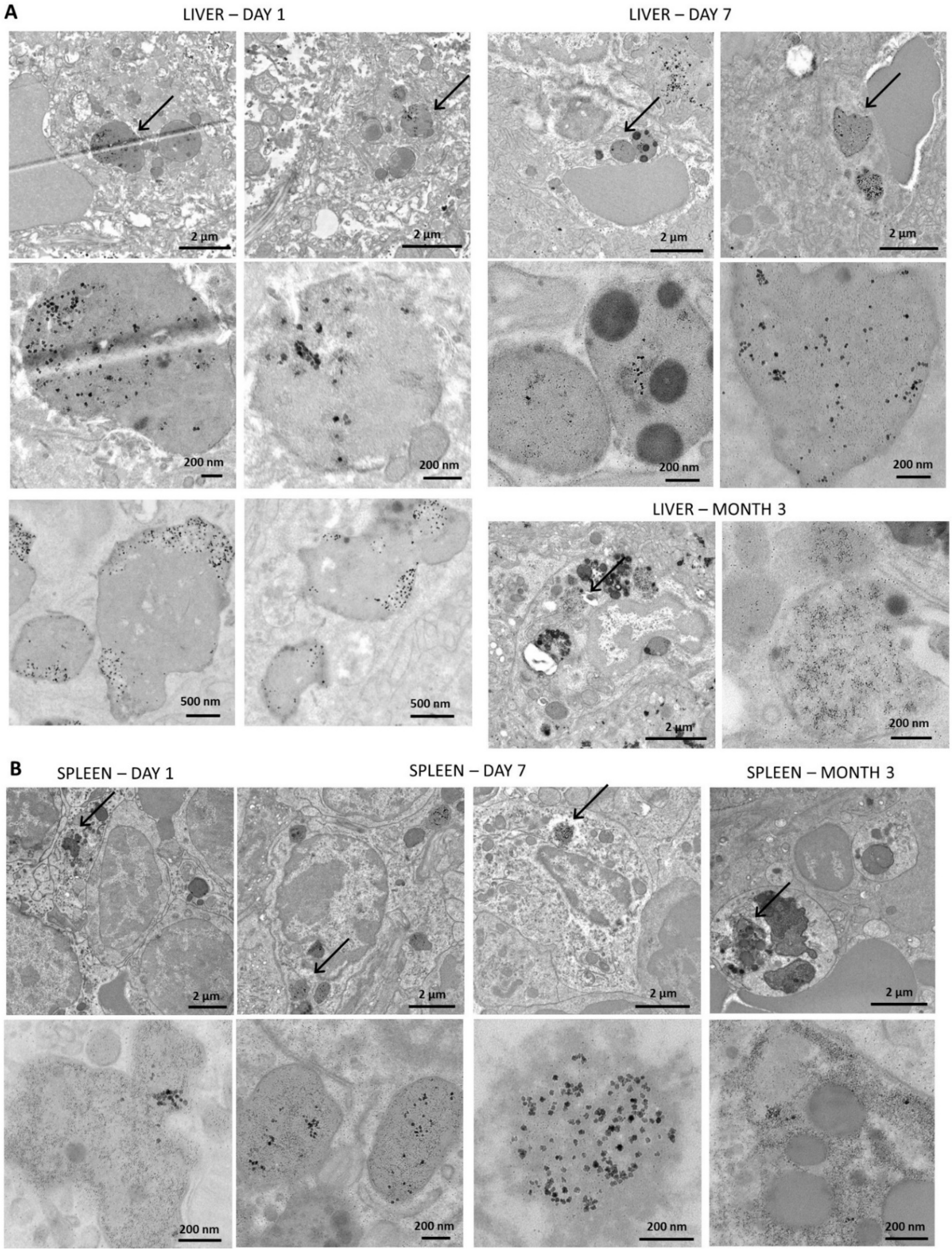

Figure S6. TEM images of the liver (**A**) and the spleen (**B**) of mice injected with 70 μg_{Fe} from iron oxide nanoflowers, at day 1, day 7 and month 3. Large fields are shown on the left, with areas indicated by a black arrow then zoomed in below or to the right.

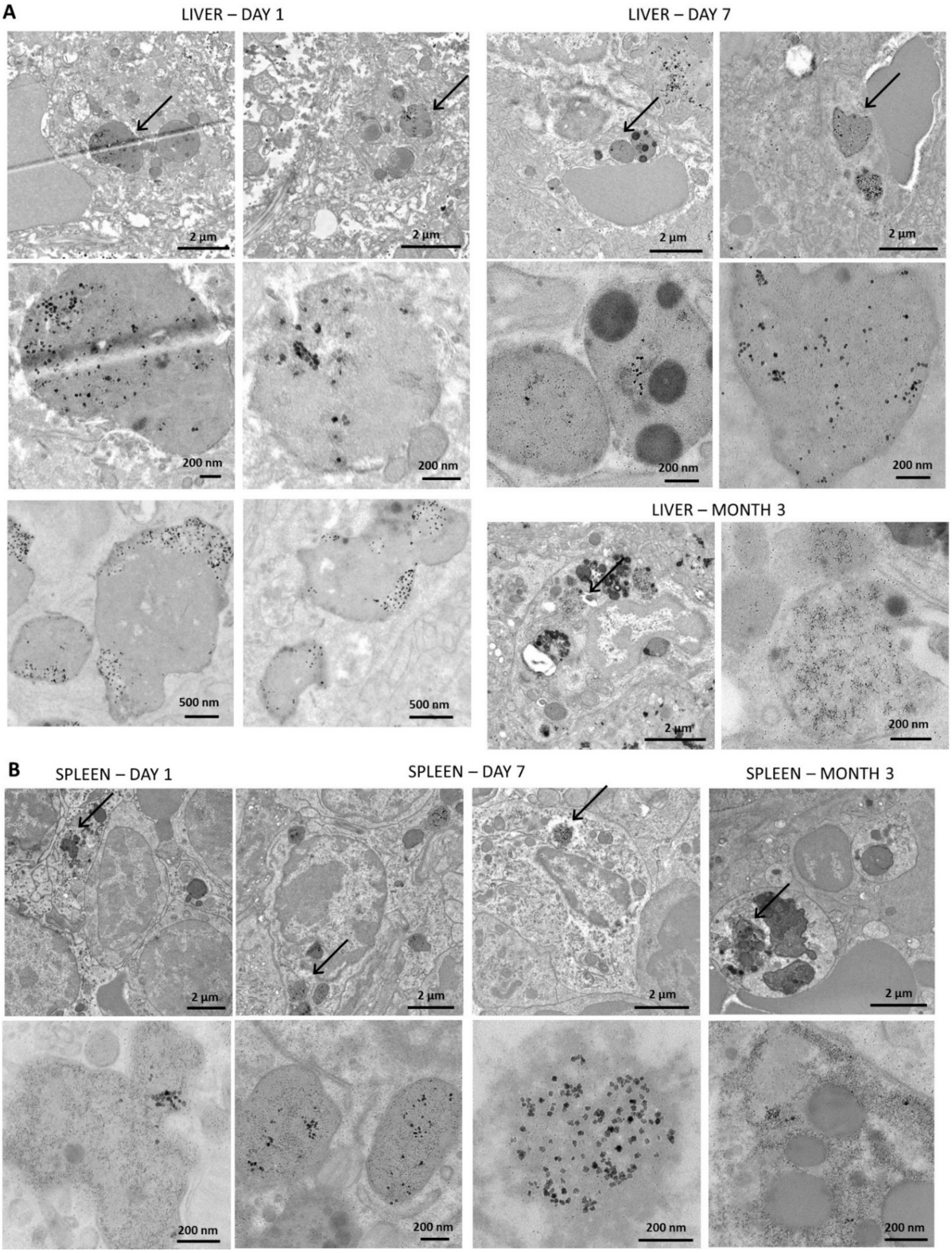

Figure S7. TEM images of the spleen (**A**) and the liver (**B**) of mice injected with 110 μg_{Cu}/20 μg_{Fe} from iron oxide @ CuS NPs, at day 1, day 7 and month 3. Areas indicated with a black arrow are zoomed within the image below (or on the right for the liver – Month 3 condition).

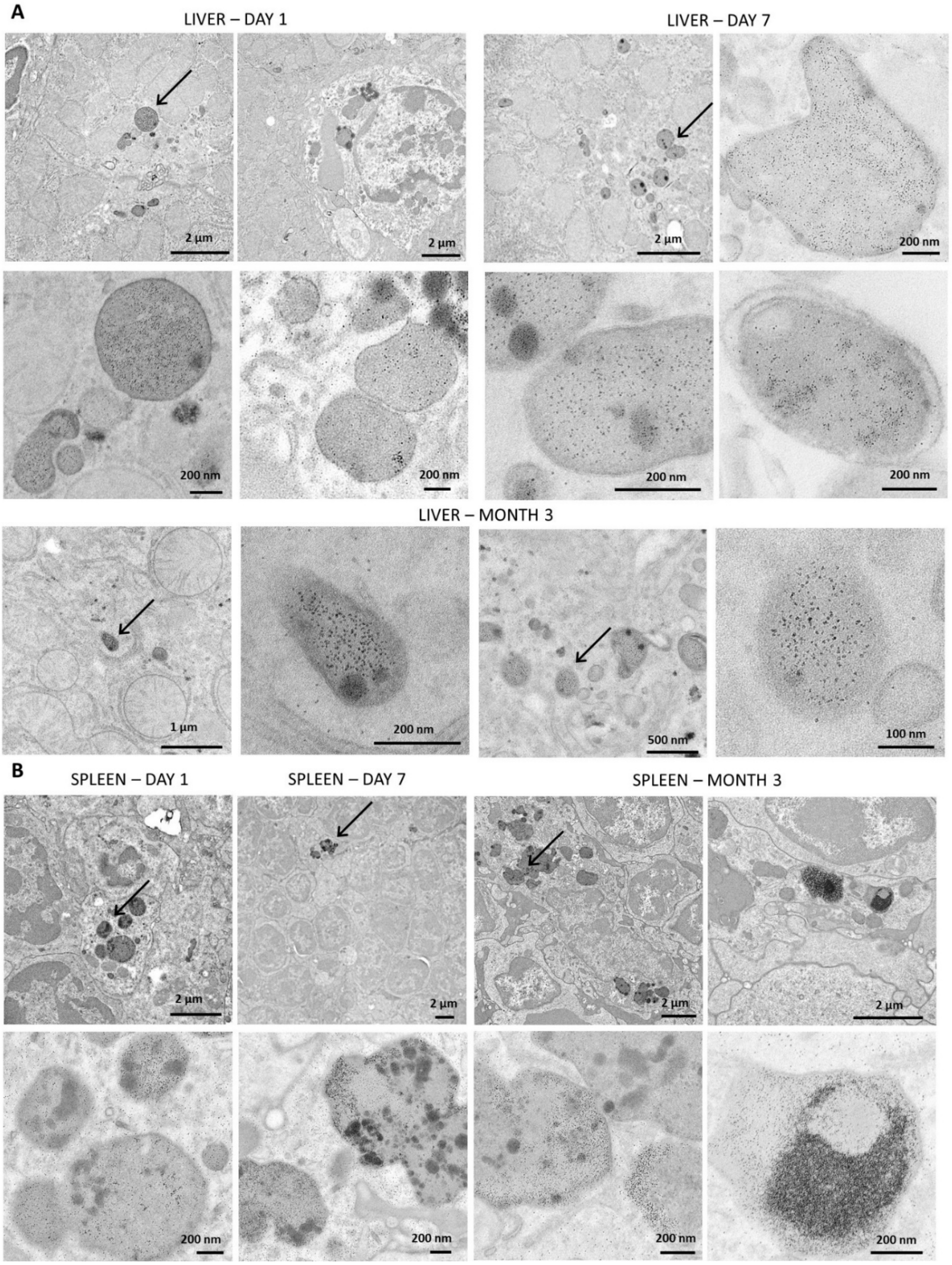

Figure S8. TEM images of the spleen (**A**) and the liver (**B**) of mice injected with 80 μg_{Cu} from CuS NPs, at day 1, day 7 and month 3. Areas indicated with a black arrow are zoomed within the image below (or on the right for the liver – Month 3 condition).
